# Concordance of Fathers and Mothers in the Assessment of Their 5-Year-Old Child’s Dental Fear

**DOI:** 10.3390/dj12030053

**Published:** 2024-02-27

**Authors:** Sanna Seppänen, Kukka Vuorenmaa, Auli Suominen, Mika Ogawa, Vesa Pohjola, Kari Rantavuori, Hasse Karlsson, Linnea Karlsson, Satu Lahti

**Affiliations:** 1 Department of Community Dentistry, University of Turku, 20014 Turku, Finland; sanna.m.seppanen@utu.fi (S.S.); kukka.u.vuorenmaa@utu.fi (K.V.); auli.suominen@utu.fi (A.S.); mika.ogawa@utu.fi (M.O.); vesa.pohjola@utu.fi (V.P.); 2 FinnBrain Birth Cohort Study, Department of Clinical Medicine, Turku Brain and Mind Center, University of Turku, 20014 Turku, Finland; kari.rantavuori@utu.fi (K.R.); hasse.karlsson@utu.fi (H.K.); linnea.karlsson@utu.fi (L.K.); 3 Department of Pediatric Dentistry and Orthodontics, University of Turku, 20014 Turku, Finland; 4 Cleft Palate and Craniofacial Center, Department of Plastic Surgery, Helsinki University Hospital and Helsinki University, 00029 Helsinki, Finland; 5 Centre for Population Health Research, University of Turku and Turku University Hospital, 20014 Turku, Finland; 6 Department of Psychiatry, University of Turku and Turku University Hospital, 20014 Turku, Finland; 7 Department of Clinical Medicine, Unit of Public Health, University of Turku, 20014 Turku, Finland; 8 Department of Clinical Medicine, Paediatrics and Adolescent Medicine, University of Turku and Turku University Hospital, 20014 Turku, Finland

**Keywords:** dental fear, dental treatment, treatment procedure, mother, father, children

## Abstract

The aim of this study was to evaluate the concordance of parents’ assessments of their child’s dental fear. Cross-sectional secondary analysis used data from the multidisciplinary FinnBrain Birth Cohort Study. Child dental fear was assessed at age 5 with the Finnish translation of the modified Children’s Fear Survey Schedule Dental Subscale (CFSS-M) by both fathers (n = 588) and mothers (n = 1100). Reply alternatives were from 1 = not afraid to 5 = very afraid and 6 = no experience coded as missing and 1. In total, 514 mother–father pairs were eligible for the analyses. Descriptive statistics, percentage agreement and Cohen’s Kappa coefficients were used in the analyses. The concordance of parents’ assessments was poor (Kappa range 0.072–0.258). The majority of parents replied “No Experience” to items related to invasive treatment or being unable to breathe. Thus, coding of this reply alternative had a significant impact on the mean values of the child’s fear. When assessing the fear of a five-year-old child, it might not be safe to rely only on one parent’s assessment, and whether or not the child has experience with the question asked should also be considered.

## 1. Introduction

Dental fear of children is relatively common, and it is known that parental dental fear correlates positively with the fear of their child [1,2,3,4,5,6,7]. When studying the fear of children, parents are often used as a proxy for the child—especially in the case of small children who may not be able to respond to surveys assessing their own fear. In most studies, the fear of the child is assessed by the mother accompanying the child to the oral health appointment [3,4].

When assessing the fear of the child, the assessments of the parent and the child themselves are not always identical. Studies have shown poor agreement between parental rating and children’s self-ratings of child dental fear [8,9,10], and that parents and children cannot reliably recognize each other’s dental fear [11,12]. In addition, parents do not necessarily assess similar matters related to their child, such as parenting behavior, school preferences, interaction with children and expectations for the children’s future [13,14,15,16,17].

Only a few studies have reported fathers’ assessments of the fear of their child or the fathers’ fear and its effect on children’s fear [9,10,18,19,20,21]. When studying the association between a parent’s and a child’s fear, the strength of the association varied for mothers and fathers [22]. This might reflect the fact that while both father and mother play an important role, their roles may differ. For example, in oral health appointments, the effect of the accompanying parent on the child might differ, thus passing the dental fear to the child differently. Therefore, it is also important to consider the roles of both parents in assessing their child’s dental fear.

However, we found only one study that looked at the concordance of mothers’ and fathers’ assessment of their child’s dental fear. The study was carried out on children aged 4–12 and it found no difference between parents’ assessment of the child’s fear and that both parents overestimated the fear of a child [5]. Thus, there is a need for more research on this topic.

The aim of this study was to evaluate the concordance of fathers’ and mothers’ assessments of the child’s dental fear in general and in different aspects of oral health care.

## 2. Materials and Methods

This was a cross-sectional secondary analysis using data collected as part of the FinnBrain Birth Cohort study (finnbrain.fi), a multidisciplinary study on the effects of environmental and genetic factors on child brain development and health [23]. Participants were mothers and their partners who were recruited after ultrasonography appointments that are offered free of charge for around gestational week 12 for every pregnant mother in Finland. Recruitment took place in municipal maternity clinics in the Hospital District of Southwest Finland during 2011–2015. Mothers were asked to invite partners (later called fathers), who did not attend the ultrasonic appointment, to participate in the study. Of those informed about the study (N = 5970), 3808 (66%) mothers and 2623 fathers, or other partners of the mother, who were expecting 3837 children (twins included), agreed to participate. Of those who agreed, 3095 (81%) mothers and 2011 (77%) fathers returned the baseline questionnaire and started the study [17]. Both parents provided written informed consent. The Ethics Committee of the Hospital District of Southwest Finland approved the study protocol (14.6.2011 ETMK:57/180/2011 § 168).

Child dental fear was measured at 5 years of age with the Finnish translation of the modified Dental Subscale of the Children’s Fear Survey Schedule CFSSM, filled out by both the father and the mother separately. Dental fear of the children was assessed as part of the questionnaire that contained also questions on psychological wellbeing, family circumstances and child-related issues such as temperament and sleep. The modified CFSS-M is found to be reliable and valid in the Finnish child and adolescent population [24].

The modified CFSS-M questionnaire evaluates a child’s dental fear level in 11 scenarios: in general, when keeping the mouth open, the dentist, teeth being cleaned by a dentist or an assistant, drilling, local anesthesia, hearing the sound of drilling, being unable to breathe, instruments put in the mouth, suction used in the mouth and dental treatment causing pain. The questions had 5-point Likert-scale reply alternatives from 1 = not afraid to 5 = very afraid and an alternative, 6 = no experience of this particular matter.

Mothers and fathers who had both filled out the modified CFSS-M, evaluating their child’s dental fear level and reporting their own dental fear three years prior to their child being measured, were selected for the study, resulting in 514 mother–father pairs. This is 16.6% of the total cohort. The responses were given about 281 (54.7%) boys and 233 (45.3%) girls.

Frequencies and descriptive statistics (mean, median, standard deviation) were calculated for each question separately for both mothers and fathers. In addition, the combined assessment of both parents was determined by calculating the mean of their responses. When calculating the average means for mothers and fathers combined, only those cases where the response of neither mother nor father was missing were included. The answers of mothers and fathers were compared using cross tabulation. All the analyses were performed with answer option 6 (no experience) coded as both 0 and 1. The concordance of the answers between mothers and fathers was assessed using percentage agreement and Cohen’s Kappa coefficient. The criteria of the interpretation of Cohen’s Kappa coefficient are as follows: values ≤ 0 as indicating no agreement; 0.01–0.20 as none to slight; 0.21–0.40 as fair; 0.41–0.60 as moderate; 0.61–0.80 as substantial; and 0.81–1.00 as almost perfect agreement [25]. Statistical analyses were conducted using IBM SPSS Statistics v. 29.00 (SPSS Inc., Chicago, IL, USA).

## 3. Results

Among the 514 mother–father pairs included in this study, the mean age of mothers at the point of birth was 31.0 years (SD 4.2) and the mean age of fathers was 32.9 years (SD 5.0). The percentage distribution of the responses of mothers and fathers to each of the fear items is given in Table 1. Slightly over half of the children were estimated to have at least some dental fear in general. For over half of the items, most parents reported “No Experience”, especially for invasive procedures such as drilling and local anesthesia. More than half of fathers and mothers responded that their child was not afraid of keeping their mouth open, and fathers reported an absence of fear more often than mothers. One in three fathers and one in four mothers responded that their child was not afraid of teeth being cleaned by a dentist or assistant. However, one in three mothers and four in five fathers reported that their child did not have experience in having their teeth cleaned. Two-thirds of the parents reported that their child had experience of having instruments put in the mouth, and one-third of the parents reported that their child was not afraid of that.

The results in Table 2 show the inter-rater agreement as percentage agreement and Kappa values. The concordance of parent’s assessment was poor, indicating that the answers of mothers and fathers differed from each other. Especially in questions where most replies were “No Experience”, the assessments of mothers and fathers differed from each other. When “No experience” was included and coded as 1, only one item, “Keeping the mouth open” and the Kappa value indicated fair agreement, while for other items, the Kappa values indicated none to slight agreement. When “No Experience” was excluded, five items reached fair agreement, while others had only none to slight agreement.

**Figure 1:**
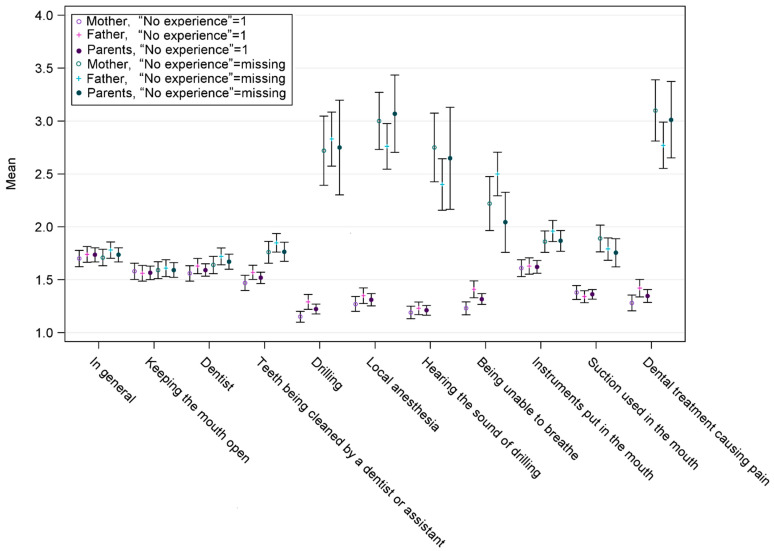
Mean values and standard deviations of the assessments of mothers, fathers, and assessments of both combined. In columns marked in blue, “No Experience” was coded as 1, and in columns marked in red, “No Experience” was excluded from the analyses.

Figure 1 shows the mean values and standard deviations for the assessments of mothers, fathers and assessments of both combined with “No Experience” responses coded in two ways. The detailed means, standard deviations and medians are presented in Table A1, Table A2, Table A3 and Table A4. When “No Experience” was excluded from the data (Table A1), the number of responses was small, especially on questions on drilling, local anesthesia, hearing the sound of drilling, being unable to breathe and dental treatment causing pain, reflecting the number of invasive procedures experienced. On the other hand, the medians of the responses of both mothers and fathers were 2 of 3, and the means were between 1.8 and 3.0. When children with “No Experience” of the items were not included, mothers rated the fear of pain, local anesthesia and hearing the sound of drilling higher than fathers, while fathers rated fear of being unable to breathe higher than mothers did.

Table A2 shows that when “No Eexperience” was included as 1, the median responses differed only for dental fear in general mothers reporting 1 point lower median than fathers. In addition, the means of the responses of both mothers and fathers were between 1.2 and 1.7. When the response option “No Experience” was included, the mean values of children’s dental fear rated by mothers and fathers were the same in six items. Fathers rated children’s dental fear higher in four items and lower in one item than mothers did.

When mothers’ and fathers’ ratings for children’s dental fear were analyzed together and those with “No Experience” were not included, the means indicating dental fear of invasive treatment were much higher than when those with “No Experience” were included in the analyses. For instance, the mean for the item “Dental treatment causing pain” was more than two times higher when those with “No Experience” were not included than when they were included (Table A3 and Table A4).

When looking at the mean sums of the mothers and fathers, Table A3 shows the responses with “No Experience” excluded. This affected markedly the n of the answers included, n being as small as 16–35 on the questions of invasive procedures. In these same questions, the means were higher than in all other questions; but then again, the standard deviations were larger due to smaller numbers of responses.

From Table A4, it can be seen that when “No Experience” was included, the mean sums of the mothers and fathers were slightly smaller for the questions on invasive procedures than for all other questions. In addition, the standard deviations of the mean sums did not differ much from the other questions.

The mean values for the different codings of the “No Experience” were rather similar when assessing the fear of children in general and for responses about keeping the mouth open, fear of the dentist, teeth being cleaned at the appointment, instruments put in the mouth and suction used in the mouth. However, the means differed considerably for responses about drilling, local anesthesia, hearing the sound of drilling, being unable to breathe and dental treatment causing pain.

## 4. Discussion

The concordance of parents’ assessments of a child’s dental fear, which this study aimed to assess, was poor. However, when looking at the means of the answers of both parents, the assessments of mothers and fathers were not substantially different from each other. In addition, the coding of the answer “No Experience” had a significant impact on the findings of the child’s fear.

In this study, mothers’ and fathers’ assessments of their child’s fear differed from each other. However, the values were often close to each other, for example, 2 and 1, instead of 4 and 1. Mothers gave either very low or high values more often than fathers, whose answers tended to be more in the middle. A considerable number of parents assessed that their child did not have any experience with the item, especially for items concerning more invasive procedures, such as drilling, rendering the sample size for these items rather small. This might affect the validity of the results.

As such, the findings were not aligned with the one study we found on the concordance of parents’ assessment of their child’s fear, which found no difference between mothers and fathers [18]. On the other hand, this study looked at the items of CFSS-M separately, instead of the sum value of CFSS-M. We came to this approach because of the relatively large proportion of “No Experience” responses. Apart from that, it is important to note that the mean values of the parent’s assessments were not statistically different from each other.

Children who are known to have dental fear usually have high levels of it [26]. The findings suggest that the fear levels did not differ considerably from those of 6-year-olds in 1998 and 2001 in dental treatment, in general, when keeping the mouth open, the dentist and teeth being cleaned by a dentist or an assistant, but differed somewhat in drilling, local anesthesia, hearing the sound of drilling and being unable to breathe [24]. The difference is likely because “No Experience” was coded as 1 in the previous study [26]. When a child does not have experience in something, they might be considered to have no fear, even though the fear may develop later.

The findings indicate that, especially for items 5–8 (drilling, local anesthesia, hearing the sound of drilling, being unable to breathe) and 11 (dental treatment causing pain), the parents most commonly answered “No Experience”. This could be explained by the fact that 66% of children at this age in Finland are caries-free, and thus do not have experience in these [27]. On the other hand, those children whose parents knew about their fear were at least somewhat afraid. If the child does not have experience with a treatment procedure, it is possible that the child is assessed as having little fear, although the fear may increase later when experiences with oral health treatment accumulate.

Several different methods were used to analyze the responses. Each method has its strengths and weaknesses. When “No Experience” was coded as 1, it affected the mean. When “No Experience” was not included, it greatly affected the number of participants included in the analyses. This is significant, as “No Experience” has been coded as 1 in previous studies [24,28] using a Likert scale of 1–5, or previous studies have not included information about the handling of missing values [5,8,18].

It is important to note that the combined answers of both parents were calculated from different data than the answers of mothers or fathers alone. When calculating the combined answers of mothers and fathers, only those cases where each question was answered were included. This explains why the means of the combined answers may be higher or lower than those of mothers or fathers only.

When assessing the fear of a five-year-old child, the role of the parent is essential because it is still difficult for the child to assess their own fear. In Finland, both parents act as caregivers for their children [29] and fathers can be important role models, especially for boys [30]. Either parent can accompany the child to the oral health treatment visit. Thus, it is important for the dental clinician to consider the importance of the different roles of the parents in the evaluation of the child’s fear.

The strength of this study is the large sample representing the general population in the geographic area [23] and the use of valid measures of dental fear [24]. The limitation of this study was that the dental fear of parents was measured when the children were two years old, whereas the assessments of the fear of their children were measured three years later. As dental fear has been shown to change over time in this population [31] and also in other populations [32], evaluation of the effect of parents’ dental fear in the assessment would not have been reliable. In the future, it would be important to study this effect.

To conclude, when assessing the dental fear of a five-year-old child, it might not be safe to rely only on one parent’s assessment. In addition, it is important to choose a correct measure and variable according to the situation. When interpreting the results, it is essential to consider the factors that affect the mean of the responses. It is important to consider how many of the respondents have experience concerning the question asked.

## Figures and Tables

**Table 1:** Percentage distribution of the responses of fathers (F) and mothers (M) to each CFSS-M item.

Item			Not Afraid	A Little Afraid	Afraid to Some Degree	Quite Afraid	Very Afraid	No Experience	Missing
		n	%	%	%	%	%	%	%
In general	M	514	49.4	32.7	12.3	1.6	1.9	2.1	0
	F	514	42.8	33.3	14.2	3.5	0.4	5.8	0
Keeping the	M	513	58.6	25.3	7.8	2.9	1.9	3.3	0.2
mouth open	F	512	53.7	24.7	8.4	3.1	1.2	8.6	0.4
Dentist	M	514	47.9	26.8	9.1	1.6	1.6	13	0
	F	514	41.2	32.3	9.3	3.1	0.6	13.4	0
Teeth being cleaned by a	M	513	30.4	20.8	7.2	1.8	1.6	38.1	0.2
dentist or assistant	F	514	26.1	27.6	11.3	1.8	0.4	32.9	0
Drilling	M	510	1.8	1.6	3.5	1.8	0.4	90.3	0.8
	F	513	2.3	4.1	4.5	3.7	1.2	84.0	0.2
Local	M	511	1.9	1.8	5.6	2.9	1.4	85.8	0.6
anesthesia	F	511	3.1	4.3	7.6	3.5	1.2	79.8	0.6
Hearing the sound of	M	510	2.3	1.9	3.7	1.9	1.0	88.3	0.8
drilling	F	512	4.3	4.7	4.1	2.7	0.4	83.5	0.4
Being unable	M	511	7.2	4.7	3.1	2.3	1.2	80.9	0.6
to breathe	F	511	7.2	7.0	6.4	4.5	1.8	72.6	0.6
Instruments put in the	M	510	31.3	24.9	8.9	3.5	1.8	28.8	0.8
mouth	F	510	22.2	30.2	8.0	3.5	1.6	33.9	0.8
Suction used	M	510	17.3	15.8	7.0	1.8	0.8	56.6	0.8
in the mouth	F	511	17.5	18.3	5.6	1.4	0	56.6	0.6
Dental treatment	M	508	1.8	2.3	3.5	4.1	1.6	85.6	1.2
causing pain	F	513	3.5	7.4	6.6	3.1	2.9	76.3	0.2

**Table 2:** The percentage (%) agreement and Kappa values for the concordance of the assessment between mothers and fathers; and the proportions where one parent reported more fear than the other. Results are presented for the two codings of the “No Experience” option: A = no experience included and coded as 1; B = no experience excluded.

Item	n		% Agreement		Kappa		% Father > Mother		% Mother > Father	
	A	B	A	B	A	B	A	B	A	B
In general	514	475	45.7	49.1	0.178	0.192	32.1	28.8	22.2	22.1
Keeping the mouth open	512	455	51.4	56.9	0.203	0.229	28.9	23.7	19.7	19.3
Dentist	514	397	40.3	47.4	0.133	0.131	33.3	30.5	26.5	22.2
Teeth being cleaned by a dentist or assistant	513	236	40.4	50.4	0.181	0.224	29.8	30.5	29.8	19.1
Drilling	509	16	79.8	43.8	0.120	0.258	6.9	31.3	13.4	25.0
Local anesthesia	508	29	74.6	37.9	0.151	0.154	9.3	24.1	16.1	37.9
Hearing the sound of drilling	508	17	77.6	41.2	0.102	0.213	9.3	47.1	13.2	11.8
Being unable to breathe	508	35	63.4	31.4	0.072	0.058	15.4	51.4	21.3	17.1
Instruments put in the mouth	506	247	34.0	45.7	0.115	0.183	38.1	32.4	27.9	21.9
Suction used in the mouth	507	110	46.5	50.9	0.126	0.231	26.0	21.8	27.4	27.3
Dental treatment causing pain	507	28	70.8	35.7	0.114	0.163	9.5	28.6	19.7	35.7

## Data Availability

Data are not available due to restrictions related to privacy and ethical issues.

## Appendix A

**Table A1:** Mean values, standard deviations and medians of the assessments, “No Experience” not included.

When Response Option ”No Experience” Was Not Included								
	Mothers				Fathers			
Item	n	Mean	SD	Median	n	Mean	SD	Median
In general	503	1.7	0.89	1	484	1.8	0.86	2
Keeping the mouth open	496	1.6	0.91	1	468	1.6	0.86	1
Dentist	447	1.6	0.87	1	445	1.7	0.85	2
Teeth being cleaned by a dentist or assistant	317	1.8	0.95	2	345	1.8	0.83	2
Drilling	46	2.7	1.13	3	81	2.8	1.17	3
Local anesthesia	70	3.0	1.16	3	101	2.8	1.11	3
Hearing the sound of drilling	56	2.8	1.24	3	83	2.4	1.13	2
Being unable to breathe	95	2.2	1.27	2	138	2.5	1.23	2
Instruments put in the mouth	362	1.9	0.99	2	336	2.0	0.94	2
Suction used in the mouth	219	1.9	0.95	2	220	1.8	0.79	2
Dental treatment causing pain	68	3.1	1.22	3	121	2.8	1.22	3

**Table A2:** Mean values, standard deviations and medians of the assessments, “No Experience” included.

When Response Option ”No Experience” Was Included (6 = 1)								
	Mothers				Fathers			
Item	n	Mean	SD	Median	n	Mean	SD	Median
In general	514	1.7	0.9	1	514	1.7	0.9	2
Keeping the mouth open	513	1.6	0.9	1	512	1.6	0.9	1
Dentist	514	1.6	0.8	1	514	1.6	0.8	1
Teeth being cleaned by a dentist or assistant	513	1.5	0.8	1	514	1.6	0.9	1
Drilling	510	1.2	0.6	1	513	1.3	0.8	1
Local anesthesia	511	1.3	0.8	1	511	1.3	0.9	1
Hearing the sound of drilling	510	1.2	0.7	1	512	1.2	0.7	1
Being unable to breathe	511	1.2	0.8	1	511	1.4	0.9	1
Instruments put in the mouth	510	1.6	0.9	1	510	1.6	0.9	1
Suction used in the mouth	510	1.4	0.8	1	511	1.3	0.6	1
Dental treatment causing pain	508	1.3	0.8	1	513	1.4	1.0	1

**Table A3:** Mean sums, standard deviations and medians, “No Experience” not included.

When Response Option ”No Experience” Was Not Included				
Mean Sum of Mothers and Fathers				
Item	n	Mean	SD	Median
In general	475	1.7	0.76	1.5
Keeping the mouth open	455	1.6	0.78	1.5
Dentist	397	1.7	0.726	1.5
Teeth being cleaned by a dentist or assistant	236	1.8	0.716	1.5
Drilling	16	2.8	0.916	2.8
Local anesthesia	29	3.1	1.01	3.0
Hearing the sound of drilling	17	2.6	1.01	3.0
Being unable to breathe	35	2.0	0.86	2.0
Instruments put in the mouth	247	1.9	0.78	1.5
Suction used in the mouth	110	1.8	0.71	1.5
Dental treatment causing pain	28	3.0	0.98	3.0

**Table A4:** Mean sums, standard deviations and medians, “No Experience” included.

When Response Option ”No Experience” Was Included 6 = 1				
Mean Sum of Mothers and Fathers				
Item	n	Mean	SD	Median
In general	475	1.7	0.75	1.5
Keeping the mouth open	512	1.6	0.76	1.5
Dentist	512	1.6	0.68	1.5
Teeth being cleaned by a dentist or assistant	513	1.5	0.63	1.5
Drilling	509	1.2	0.53	1.0
Local anesthesia	508	1.3	0.67	1.0
Hearing the sound of drilling	508	1.2	0.52	1.0
Being unable to breathe	508	1.3	0.59	1.0
Instruments put in the mouth	506	1.6	0.69	1.5
Suction used in the mouth	507	1.4	0.53	1.0
Dental treatment causing pain	507	1.3	0.69	1.0
